# An IoMT-Enabled Smart Healthcare Model to Monitor Elderly People Using Machine Learning Technique

**DOI:** 10.1155/2021/2487759

**Published:** 2021-11-25

**Authors:** Muhammad Farrukh Khan, Taher M. Ghazal, Raed A. Said, Areej Fatima, Sagheer Abbas, M. A. Khan, Ghassan F. Issa, Munir Ahmad, Muhammad Adnan Khan

**Affiliations:** ^1^School of Computer Science, National College of Business Administration and Economics, Lahore 54000, Pakistan; ^2^Lahore Institute of Science and Technology, Lahore 54792, Pakistan; ^3^Center for Cyber Security, Faculty of Information Science and Technology, University Kebangsaan Malaysia (UKM), 43600 Bangi, Selangor, Malaysia; ^4^School of Information Technology, Skyline University College, University City Sharjah, 1797 Sharjah, UAE; ^5^Canadian University Dubai, Dubai, UAE; ^6^Department of Computer Science, Lahore Garrison University, Lahore 54792, Pakistan; ^7^Riphah School of Computing & Innovation, Faculty of Computing, Riphah International University Lahore Campus, Lahore 54000, Pakistan; ^8^Pattern Recognition and Machine Learning Lab, Department of Software, Gachon University, Seongnam Gyeonggido 13120, Republic of Korea

## Abstract

The Internet of Medical Things (IoMT) enables digital devices to gather, infer, and broadcast health data via the cloud platform. The phenomenal growth of the IoMT is fueled by many factors, including the widespread and growing availability of wearables and the ever-decreasing cost of sensor-based technology. The cost of related healthcare will rise as the global population of elderly people grows in parallel with an overall life expectancy that demands affordable healthcare services, solutions, and developments. IoMT may bring revolution in the medical sciences in terms of the quality of healthcare of elderly people while entangled with machine learning (ML) algorithms. The effectiveness of the smart healthcare (SHC) model to monitor elderly people was observed by performing tests on IoMT datasets. For evaluation, the precision, recall, fscore, accuracy, and ROC values are computed. The authors also compare the results of the SHC model with different conventional popular ML techniques, e.g., support vector machine (SVM), K-nearest neighbor (KNN), and decision tree (DT), to analyze the effectiveness of the result.

## 1. Introduction

The Internet of things (IoT) is a system of interrelated computing devices that are provided with unique identifiers (UIDs) and the ability to transfer data over a network without requiring human-to-human or human-to-computer interaction. The practical application of IoT devices with medical technology used in healthcare is the IoMT. IoT helps to transfer the data of healthcare devices and applications on medical IT servers for remote analysis. IoMT allows medical staff to access patients' healthcare data remotely through a web platform or any mobile application in real time to deal with patients' medical issues and help them avoid any future severe circumstances. This technology of interconnected medical devices allows the patients to monitor their health conditions following the treatment suggestions of the doctors by engaging in smart devices and applications while creating ease for the doctors to know the medical history of the patients before the checkup through the collection of real-time data using IoMT [1].

In short, healthcare coupled with IoMT improves the quality of life, gives superior care administrations, and can make more cost-effective frameworks. In IoMT, a supportive mechanism between the sensors, communication modules, and users is required to efficiently and securely provide health services. IoMT technology is considered helpful in strengthening healthcare by providing self-care and early diagnosis features using a remote monitoring system. People are becoming more engaged and aware of their well-being as healthcare innovations advance. In this situation, the need for remote treatment is higher than ever. However, existing healthcare systems require technology to transform patient care by providing real-time patient information and encouraging doctors to take practical treatment steps [2].

Health facilities are not accessible or affordable to all, despite having excellent infrastructure and cutting-edge technology. Smart healthcare (SHC) aims to assist users by informing them about their medical conditions and keeping them informed about their health. SHC allows people to handle specific emergencies on their own. SHC employs modern information technology, e.g., IoT, big data, cloud computing, and artificial intelligence (AI), to completely change the current healthcare system into a more efficient and convenient one [3].

SHC promotes interaction among all stakeholders in the medical industry. It ensures that users receive the services they require, assisting parties in making informed decisions and facilitating resource distribution. SHC technology improves disease diagnosis, patient treatment, and overall quality of life. SHC systems based on IoT and big data can link patients with providers across various healthcare systems efficiently. SHC systems are also becoming more linked to different wearable devices used for real-time healthcare surveillance through the Internet. In SHC, wearable health devices (such as blood pressure monitors, glucometers, smartwatches, and so on) combined with IoT gadgets allow for continuous patient surveillance and treatment even when they are at home. The World Health Organization (WHO) predicts that “by 2050, the number of senior citizens will have risen to about 1.5 billion” [4]. The elderly population (including persons older than 60 years) requires the most significant healthcare needs. Elderly people are more vulnerable to chronic diseases because of a decreasing immune system and require regular visits to healthcare facilities and more hospitalizations.

Elderly people move from one level of treatment to another as they age. These patients, on the other hand, have limited access to specialized senior care facilities. A multifaceted strategy is essential to avoid health issues in the elderly population. An excellent preventive system that includes routine medical checkups allows for early disease detection and optimal care. Furthermore, older adults and their families should be aware of potential diseases to recognize them and start treatment as soon as possible [5].

Many countries built technologies and communication networks to help people live their lives more efficiently and simply. Many industries drive technology development, which transforms people's lifestyles. A related perspective on the link between technology and aging is technological transition and consumption, emphasizing the aged as active users and co-creators. Smart homes (SHs), smart cities, and mobile apps are examples of developments and innovations to assist the senior population's well-being through generic design. In the case of elderly people, IoMT-enabled SHC is an emerging solution for providing constant and holistic monitoring, reducing human caregiver effort, and assisting in clinical decision making. Rather than being hospitalized, elderly people can be supported using various “smart” devices in their own homes [6]. The SH idea is a viable and cost-effective approach to improve non-intrusive home treatment for seniors, enabling greater independence, ensuring good health, and avoiding social isolation. According to [7], SH solutions are regarded as information-based technology that gathers and disseminates user data with the resident, family members, and primary care physicians in a passive manner. SH solution also refers to using essential and assistive gadgets to create an environment where many house features are automated and devices may connect. SHs assist elderly people in their homes. Sensors and actuators integrated into the housing infrastructure track the occupants' bodily signals, ambient conditions, daily behavior patterns, and sleep patterns, among other things. SHs also have a role in improving people's quality of life. Health and wellness tracking technologies include wearable activity trackers using accelerometers and sensors. They also include non-wearable, embedded sensor activity monitors to track everyday activities.

An emergency medical service system (EMSS) is a complete system that organizes individuals, facilities, and equipment to offer health and safety services to sufferers of unexpected sickness or injury in a quick, effective, and coordinated way. EMSS aims to provide prompt treatment to sufferers of unexpected and life-threatening accidents to avoid unnecessary fatality or long-term morbidity. With advanced information and communication technologies, EMSS can deliver services that address the requirements of the elderly [8].

Elderly people having a weak immune system require daily checkups to maintain their health. For this purpose, they need to go to the hospitals or clinics, which is the main issue because of the mobility problems faced by elderly people [9]. In this case, SHC can provide the facility to the elderly by continuously monitoring their health without going to the hospital and helping medical experts make efficient decisions about patient health. Despite having this technology there exist some other challenges that restrict the usage of SHC for elderly people's health monitoring. Many senior citizens are reluctant to adopt this technology.

Many senior citizens are unfamiliar with current technology [10], its advantages, and how to use the gadgets and applications. Furthermore, many individuals lack access to training and technical assistance, and their capacity to utilize and manage technological systems is a worry. Other concerns include issues of privacy, protection, and reliability. The cost and use of communication technologies are critical elements in expanding medical facilities for the elderly to address technological challenges. Device design and usability are important requirements that should be prioritized to make the software simple to understand and use.

ML, an application of AI, gives systems the ability to learn from data automatically and make decisions without human assistance. ML is the study of allowing machines to learn and create their programs to make them more human-like in their actions and decisions. ML enables the systems to improve them from experience without being explicitly programmed. It also enables the machines or software to analyze, predict, and sort huge amounts of data. To make better decisions in the future, the learning process begins with data, instructions, and observations. ML algorithms use statistics to identify the patterns in massive quantities of data, e.g., numbers, words, images, etc. ML is subdivided into supervised, unsupervised, semisupervised, and reinforcement ML that uses different types of data and produces specific results [11].

## 2. Literature Review

Many studies have been done on the emerging technology named IoMT. Researchers have published many papers from a different perspective, focusing on specific issues and challenges.

Iyer [12] proposed a framework and protocol related to an IoT-based patient monitoring system and suggested that patients' health can be monitored using IoT devices and sensors connected to the Internet. The medical nursing system (MNS) based on IoT has been designed that uses different communication methods to transfer the data, e.g., sensors, Wireless Fidelity (Wi-Fi), radio-frequency identification (RFID), and Bluetooth [13]. In [14], the authors proposed the patient monitoring system with the help of patient body weight. Another IoT-based system was proposed in [15] to monitor and track autism patients with the help of sensors by collecting the signals from the brain.

A new scheme based on IoT was proposed in [16] to convert the old hospitals into smart hospitals that will help manage the information in an advanced way. Another design using IoT as a back-end platform was proposed in [17] to monitor the health of aged patients by a method of the end-to-end medical healthcare system. An Indian researcher [18] presented a model based on IoT for the electronic healthcare unit using RFID technology and an experienced healthcare system Mycin (an AI-based system to identify bacteria causing different infections).

Pinto [19] presented a novel idea in cardio signals and proposed a technique for cardiac patients named Inexpensive Cardiac Arrhythmia Management (iCarMa) that will indicate the severity of the cardiac patient and its timely detection and diagnosis. Research has been conducted to continuously monitor the patient's health using an accurate algorithm for sensing the patient's events, e.g., steps counting, immobility and fall, etc.

A fascinating idea presented in [20] was to help non-professionals know about the disease with the help of a bot. This bot can be linked with various sensors on a smartphone to give a more flexible service utilizing IoMT. An emphasis on its use in [21] is that it will be very beneficial for the patients if major diseases can be predicted early on. This can be done with the help of IoT that facilitates the patients in the domain of remote healthcare systems.

Papageorgiou [22] proposed cost-efficient IoT-based living assistance for elderly people. It tracks and stores the critical details of patients employing a cloud-connected wristband. This proposed scheme triggered an alarm during emergency conditions to assist patients by alerting the healthcare experts to take the appropriate steps and decisions. Darshan [23] presented a healthcare monitoring system that offers emergency assistance to the patients through assessing their emergency condition based on their movement monitoring.

This paper reports a practical cryptosystem to secure the transmission of medical images in an Internet of Healthcare Things (IoHT) environment. The dynamics of a 2D trigonometric map created utilizing various well-known maps, such as logistic-sine-cosine maps, are investigated in this research. The map has an endless number of solutions, according to a stability study. The map's complicated dynamics are demonstrated using the Lyapunov exponent, bifurcation figure, and phase portrait. A strong cryptosystem is built using the map's sequences. First, the newly developed trigonometric map generates three key streams combined with the picture mechanisms (R, G, and B) to calculate the Hamming distance. The output distance vector, conforming to each section, is then Bit-XORed with each of the critical streams. The subsequent shuffled vectors are then Bit-XORed (diffusion) by the saved outputs as of the early stage, and finally, the image vectors are joined to create the encrypted image. The data stored in the system must be encrypted or anonymized using cryptography and data anonymization techniques [24].

Tamper-proof steganography includes efficient procedures to encrypt the image or concealed message before implanting it. Quantum inspired varients of controlled alternative quantum walks (CAQWs) which are used to determine the pixels for secret/hidden bits in the carrier image. The design employed in our method prevents the need for pre or post-encryption of the carrier and secret images. Also, our design shortens removing hidden material since only the stego image and primary conditions to run the CAQWs are required. The designed protocol showed extraordinary outcomes in terms of their security, good visual quality, high resistance to data loss bouts, and high embedding capacity [25].

In quantum computing, a quantum algorithm is an algorithm which runs on a realistic model of quantum computation, the most commonly used model being the quantum circuit model of computation. Quantum walks establish a universal quantum computational model extensively utilized in cryptography. This research designed a new encryption appliance for privacy-preserving IoT-based healthcare schemes to defend patients' privacy. The encryption/decryption procedures are based on measured alternate quantum walks. The simulation results show that image encryption protocol is healthy and well organized for protecting patients' privacy protection [26]. Another healthcare monitoring system was proposed in [27] that is based on lightweight sensor-enabled wearable devices. These devices collect, analyze, and share real-time patient healthcare information. In this model, data are collected using an “Arduino-based wearable system” with body sensor networks. Also, “LabVIEW” is integrated with this system to allow for remote patient surveillance.

In [28], a proposed system includes feasible IoT wearable devices to collect the patient's health-related information. It used different ML-based classification methods such as “DT, logistic regression (LR), and library for support vector machine (LibSVM)” to forecast the occurrence of illnesses. Lastly, a “mathematical model” is utilized to propose a personalized IoT solution for each scenario.

Ghose [29] proposed an ML algorithm that uses the Naive Bayes (NB) algorithm and the SVM to detect and analyze heart disease. To predict coronary heart disease, SVM and Bayes Net algorithms were used [30, 31].

Various ML methods are used to improve the accuracy of diabetic disease inspection. The authors suggested an ML algorithm that uses NB [32] and DT [33] to predict diabetic disorders. Otoom [34] used ML algorithm classification and regression tree (CART) to help diagnose diabetes.

ML methods are also used to forecast thyroid diseases. SVM and DT are employed as classification algorithms, with the dataset coming from the UCI repository. Advanced methods for thyroid disease diagnosis proposed in [35] use fuzzy maps and data mining algorithms.

A wireless body sensor network (WBSN)-enabled IoT healthcare solution was proposed in [36, 37]. It uses a wireless body network made up of small, lightweight sensor nodes to keep track of the patient. This solution employs a variety of ML techniques to improve security by protecting WBSN from hackers.

## 3. Effectiveness Comparison of IoMT-Enabled Smart Healthcare Model

In this research work, a model is developed to monitor elderly people's activities and provide them automated assistance when required. The IoMT has a vital role to play in monitoring elderly people. In this model, an artificial neural network (ANN) approach is used to monitor elderly people intelligently and efficiently.

**Figure 1:**
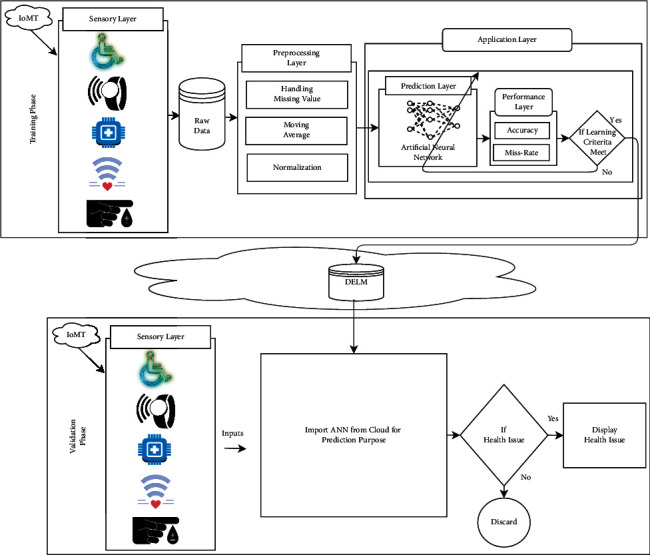
IoMT-enabled smart model to monitor elderly people's health using ML.

Figure 1 demonstrates the IoMT-enabled SHC model, which depends on two phases: training and validation. The cloud is used for communication between the two phases. The training phase is composed of the three layers: the sensory layer, preprocessing layer, and application layer. The sensory layer contains various input parameters that get the human body's values and pass these values through IoMT to store in a database. The data received through IoMT might hold missing or noisy data. So, they are known as raw data. The next layer is the preprocessing layer. It is an important layer that handles the missing values by moving average and normalization to remove the noisy data. After this process, the preprocessing layer's output is sent to the application layer. The application layer is further divided into two layers, namely, prediction layer and performance layer.

In the prediction layer, ANN is utilized to predict the disease further. It consists of three layers named as input, hidden, and output layers. The backpropagation algorithm involves several steps: weight initialization, feedforward, feedback error propagation, and weight and bias updates. In the hidden layer, each neuron has a function of activation like *f*(*j*) = sigmoid(*j*).

The sigmoid function for input and the model's hidden layer can be written as

(1) $$\begin{array}{c} \psi_{j}=b_{1}+\sum_{i=1}^{m}\left(\omega_{ij}*r_{i}\right),\\ \varphi_{j}=\frac{1}{1+e^{-\psi_{j}}},\;\text{where }j=1,2,3,\ldots ,n. \end{array}$$

Input is taken from the output layer as shown in the following equation:

(2) $$\begin{array}{c} \psi_{k}=b_{2}+\sum_{j=1}^{n}\left(\upsilon_{jk}*\varphi_{j}\right). \end{array}$$

Output layer activation function is shown in the following equation

(3) $$\begin{array}{c} \varphi_{k}=\frac{1}{1+e^{-\psi_{k}}},\;\text{where }k=1,2,3,\ldots ,r. \end{array}$$

Error in backpropagation can be written as

(4) $$\begin{array}{c} E=\frac{1}{2}\sum_{k}{\left(\tau_{k}-\varphi_{k}\right)}^{2}, \end{array}$$

where *τ*_*k*_ represents the desired output and out_*k*_ is the calculated output.

In equation (5), the layer is written as the rate of change in weight for the output.

(5) $$\begin{array}{c} \varphi W\;\propto -\frac{\partial E}{\partial W},\\ \varphi \upsilon_{j,k}=-\epsilon \frac{\partial E}{\partial \nu_{j,k}}. \end{array}$$

The chain rule method is written as follows:

(6) $$\begin{array}{c} \varphi \upsilon_{j,k}=-\;ɛ\;\frac{\partial E}{\partial \varphi_{k}}\times \frac{\partial \varphi_{k}}{\partial \psi_{k}}\times \frac{\partial \psi_{k}}{\partial \nu_{j,k}}. \end{array}$$

The value of weight change can be found by inserting the values in equation (6), and the results are shown in the following equation:

(7) $$\begin{array}{c} \varphi \upsilon_{j,k}=\epsilon \left(\tau_{k}-\varphi_{k}\right)\times \varphi_{k}\left(1-\varphi_{k}\right)\times \left(\varphi_{j}\right),\\ \varphi \upsilon_{j,k}=\epsilon \xi_{k}\varphi_{j}, \end{array}$$

where

(8) $$\begin{array}{c} \xi_{k}=\left(\tau_{k}-\varphi_{k}\right)\times \varphi_{k}\left(1-\varphi_{k}\right),\\ \varphi \omega_{i,j}\propto -\left[\sum_{k}\frac{\partial E}{\partial \varphi_{k}}\times \frac{\partial \varphi_{k}}{\partial \psi_{k}}\times \frac{\partial \psi_{k}}{\partial \varphi_{j}}\right]\times \frac{\partial \varphi_{j}}{\partial \psi_{j}}\times \frac{\partial \psi_{j}}{\partial \omega_{i,j}},\\ \varphi \omega_{i,j}=-ɛ\left[\sum_{k}\frac{\partial E}{\partial \varphi_{k}}\times \frac{\partial \varphi_{k}}{\partial \psi_{k}}\times \frac{\partial \psi_{k}}{\partial \varphi_{j}}\right]\times \frac{\partial \varphi_{j}}{\partial \psi_{j}}\times \frac{\partial \psi_{j}}{\partial \omega_{i,j}},\\ \varphi \omega_{i,j}=ɛ\left[\sum_{k}\left(\tau_{k}-\varphi_{k}\right)\times \varphi_{k}\left(1-\varphi_{k}\right)\times \left(\nu_{j,k}\right)\right]\times \varphi_{k}\left(1-\varphi_{k}\right)\times r_{i},\\ \varphi \omega_{i,j}=\;\varepsilon \;\left[\sum_{k}\left(\tau_{k}-\varphi_{k}\right)\times \varphi_{k}\left(1-\varphi_{k}\right)\times \left(\nu_{j,k}\right)\right]\times \varphi_{j}\left(1-\varphi_{j}\right)\times r_{i},\\ \varphi \omega_{i,j}=\;ɛ\;\left[\sum_{k}\xi_{k}\left(\nu_{j,k}\right)\right]\times \varphi_{j}\left(1-\varphi_{j}\right)\times r_{i},\\ \varphi \omega_{i,j}=\;ɛ\xi_{j}\;r_{i}, \end{array}$$

where

(9) $$\begin{array}{c} \xi_{j}=\left[\sum_{k}\xi_{k}\left(\nu_{j,k}\right)\right]\times \varphi_{j}\left(1-\varphi_{j}\right). \end{array}$$

Output and hidden layers are shown in equation (10) in which the weight and bias between them are updated:

(10) $$\begin{array}{c} \nu_{j,k}^{+}=\nu_{j,k}+\lambda_{F}\varphi \upsilon_{j,k}. \end{array}$$

The process of updating weight and bias between the input layer and the hidden layer is shown in the following equation:

(11) $$\begin{array}{c} \omega_{i,j}^{+}=\omega_{i,j}+\lambda_{F}\varphi \omega_{i,j}, \end{array}$$

where *λ*_*F*_ is the learning rate of the IoMT-enabled smart model. The convergence of the model depends upon the careful selection of *λ*_*F*_. The equation above is used to update the weight of the hidden and input layers.

After the prediction layer, the output of the prediction layer will be sent to the performance layer to predict the healthcare issue based on accuracy and miss rate whether the learning criteria meet or not. In the case of “no,” the prediction layer will be updated, but in the case of “yes,” the output will be stored on the cloud database. Now, in the validation phase, the input will be sensed from input layer parameters and sent to ANN to determine whether the healthcare issue is found or not. In the case of “no,” the process will be discarded, and in the case of “yes,” the message will display that healthcare issue found.

## 4. Simulation Results

This paper presents a SHC system that can enable remote healthcare consultations, elderly management, and homecare over global wide area networks and heterogeneous platforms. The simulation results of the research are described below.

Tables 1 and 2 show the training and validation concerning accuracy and miss rate. ANN algorithm has been implemented to the dataset of 4848 sets of records; furthermore, 3393 samples (70%) and 1455 samples (30%) are used for training and validation purpose, respectively. In performance evaluation layer, various statistical metrics are used to measure the proposed system performance as shown in equation (12). The parameters are derived by the following formulas:

(12) $$\begin{array}{c} \text{sensitivity}=\frac{\sum \text{true positive}}{\sum \text{condition positive}},\\ \text{specificity}=\frac{\sum \text{true negative}}{\sum \text{condition negative}},\\ \text{accuracy}=\frac{\sum \text{true positive}+\sum \text{true negative}}{\sum \text{total population}},\\ \text{miss rate}=\frac{\sum \text{false negative}}{\sum \text{condition positive}},\\ \text{fallout}=\frac{\sum \text{false positive}}{\sum \text{condition negative}},\\ \text{likelihood positive ratio}=\frac{\sum \text{true positive ratio}}{\sum \text{false positive ratio}},\\ \text{likelihood negative ratio}=\frac{\sum \text{true positive ratio}}{\sum \text{false positive ratio}},\\ \text{positive predictive value}=\frac{\sum \text{true positive}}{\sum \text{predicted condition positive}},\\ \text{negative predictive value}=\frac{\sum \text{true negatiive}}{\sum \text{predicted condition negative}}. \end{array}$$

The IoMT-enabled SHC model estimates anticipated output as negative (−1) and positive (1). The consequent output of value negative (−1) indicates that a health issue is found, whereas positive (1) indicates no health issue.

Table 1 shows IoMT-enabled SHC model monitoring of elderly people during the training phase. During tranining, 1522 positive (healthy) and 1871 negative (unhealthy) samples are used. It clearly observed that 1422 samples are correctly predicted in the case of healthy samples., while 100 records are wrongly predicted as negative, implying that there is a healthcare issue. Similarly, a total of 1871 samples were picked, with negative indicating the presence of a healthcare issue. 1754 samples are correctly predicted as negative, indicating the presence of a healthcare issue. 117 samples are incorrectly predicted as positive, indicating the absence of a healthcare issue, although a healthcare issue exists.

Table 2 displays the model's prediction of health status during the validation phase. During validation, a total of 1455 samples are utilized in which 686 are positive (healthy) and 769 are negative (unhealthy) samples are used. It is discovered that 626 samples contain true positives that are accurately forecasted, and no healthcare issue is discovered, while 60 records are mistakenly predicted as negative, implying that a healthcare issue is discovered. Similarly, 769 samples were picked, with negative indicating the presence of a health issue. 710 samples are accurately predicted as negative, indicating the presence of a healthcare issue. 59 samples are incorrectly predicted as positive, indicating the absence of a healthcare issue, although healthcare issues exist.

Table 3 shows the model's accuracy, sensitivity, specificity, miss rate, and precision during the training and validation phase. The model during training gives accuracy, sensitivity, specificity, miss rate, and precision of 0.936, 0.934, 0.937, 0.064, and 0.924, respectively. During validation, the model gives accuracy, sensitivity, specificity, miss rate, and precision of 0.918, 0.912, 0.923, 0.082, and 0.913, respectively.

Furthermore, specific statistical measures of the model are included to forecast values during training, e.g., fallout, LR+, LR−, and NPV give the result of 0.062, 15.064, 0.068, and 0.946, and during validation, they are 0.076, 12, 0.088, and 0.922, respectively.

According to the results shown in Table 4, the IoMT-enabled SHC model is more effective than the conventional ML classification-based methods like SVM, KNN, and DT while analyzing the IoMT data.

The comparison of the previously published approach and the proposed model to monitor elderly people is shown in Table 5. The proposed model achieved 0.936 accuracy for monitoring the healthcare of elderly people in an efficient way which is better than the previous approaches.

## 5. Conclusion

The research is responsible for overcoming the challenges of elderly care services. The research realizes the needs of the elderly healthcare system. In this research work, innovative medical services for the elderly are compared based on the real needs and challenges of the elderly and caregivers. To meet the basic needs of elderly healthcare, the researchers used ML techniques for getting better outcomes. After simulation results, the research conclusions are summarized: the elderly healthcare service interface of the IoMT has a higher accuracy during validation, which gives accuracy, sensitivity, specificity, miss rate, and precision of 0.918, 0.912, 0.923, 0.082, and 0.913, respectively. The system of the proposed approach may be improved in the future by using a fusion-based machine learning approach and federated learning approach as well.

## Figures and Tables

**Table 1:** Training of the IoMT-enabled SHC model during the monitoring of elderly people's health.

Training results			
Input	Total number of samples (3393)	Result (output)	
	Expected output	Predicted positive	Predicted negative
	1522 (positive)	True positive (TP)	False negative (FN)
		1422	100
	1871 (negative)	False positive (FP)	True negative (TN)
		117	1754

**Table 2:** Validation of the IoMT-enabled SHC model during the monitoring of elderly people's health.

Validation results			
Input	Total number of samples (1455)	Result (output)	
	Expected output	Predicted positive	Predicted negative
	686 (positive)	True positive (TP)	False negative (FN)
		626	60
	769 (negative)	False positive (FP)	True negative (TN)
		59	710

**Table 3:** The training and validation of the IoMT-enabled SHC model using different statistical measures.

	Accuracy	Sensitivity (TPR)	Specificity (TNR)	Miss rate (FNR)	Fallout (FPR)	LR+	LR−	PPV (precision)	NPV
Training	0.936	0.934	0.937	0.064	0.062	15.064	0.068	0.924	0.946
Validation	0.918	0.912	0.923	0.082	0.076	12	0.088	0.913	0.922

**Table 4:** Effectiveness comparison of precision, recall, fscore, and accuracy of different ML-based IoMT-enabled SHC models.

	Accuracy	Sensitivity (TPR)	Specificity (TNR)	Miss rate (FNR)	Fallout (FPR)	LR+	LR−	PPV (precision)	NPV
ANN	0.936	0.934	0.937	0.064	0.062	15.064	0.068	0.924	0.946
SVM	0.89233	0.89213	0.90113	0.08523	0.07423	9.3443	0.09143	0.65432	0.90501
KNN	0.84828	0.84765	0.8513	0.08012	0.06923	8.2311	0.87213	0.84612	0.84621
Decision tree	0.83886	0.83776	0.8432	0.07933	0.06831	8.1432	0.85231	0.83531	0.83632

**Table 5:** Comparison of the proposed system with previously published approaches.

Authors	Approach	Accuracy	Miss rate
Qin et al. [38]	Logistic regression	0.672	0.328
Tang et al. [39]	XGBoost classifier	0.892	0.108
Abro et al. [40]	Random forest classifier	0.606	0.394
Dimitriadis et al. [41]	RF	0.619	0.381
Liu et al. [42]	MSDNN	0.754	0.246
Lu et al. [43]	3D ResNet	0.830	0.170
The proposed model to monitor elderly people	Artificial neural network	0.936	0.064

## Data Availability

The data used to support the findings of this study are available from the corresponding author upon request.

## References

[1] Abbas Khan T., Abbas S., Ditta A. (2020). IoMT-based smart monitoring hierarchical fuzzy inference system for diagnosis of covid-19. *Computers, Materials & Continua*.

[2] Sultan K., Naseer I., Majeed R. (2021). Supervised machine learning-based prediction of covid-19. *Computers, Materials & Continua*.

[3] Khan Q.-T.-A., Abbas S., Adnan Khan M., Fatima A., Alanazi S., Sabri Elmitwally N. (2021). Modelling intelligent driving behaviour using machine learning. *Computers, Materials & Continua*.

[4] WHO (2011). *Global Health and Aging (NIH Publication No. 11-7737)*.

[5] Ghaffar A., Alanazi S., Alruwaili M. (2021). Multi-stage intelligent smart lockdown using sir model to control covid 19. *Intelligent Automation & Soft Computing*.

[6] Aftab S., Alanazi S., Ahmad M., Adnan Khan M., Fatima A., Sabri Elmitwally N. (2021). Cloud-based diabetes decision support system using machine learning fusion. *Computers, Materials & Continua*.

[7] Inam A., Zhuli, Sarwar A. (2021). Detection of covid-19 enhanced by a deep extreme learning machine. *Intelligent Automation & Soft Computing*.

[8] Majed Alotaibi S., Atta-ur-Rahman A. U., Adnan Khan M., Khan M. A. (2021). Ensemble machine learning based identification of pediatric epilepsy. *Computers, Materials & Continua*.

[9] Hannan Khan A., Adnan Khan M., Abbas S. (2021). Simulation, modeling, and optimization of intelligent kidney disease predication empowered with computational intelligence approaches. *Computers, Materials & Continua*.

[10] Tabassum N., Ditta A., Alyas T. (2021). Prediction of cloud ranking in a hyperconverged cloud ecosystem using machine learning. *Computers, Materials & Continua*.

[11] Courtney K. L. (2008). *Needing Smart Home Technologies: The Perspectives of Older Adults in Continuing Care Retirement Communities*.

[12] Iyer A. S. (2015). Diagnosis of diabetes using classification mining techniques. https://arxiv.org/abs/1502.03774.

[13] Asthana S. A. A recommendation system for proactive health monitoring using IoT and wearable technologies.

[14] Kim S.-H., Chung K. (2015). Emergency situation monitoring service using context motion tracking of chronic disease patients. *Cluster Computing*.

[15] Jara A. J., Zamora-Izquierdo M. A., Skarmeta A. F. (2013). Interconnection framework for mHealth and remote monitoring based on the internet of things. *IEEE Journal on Selected Areas in Communications*.

[16] Chan M., Campo E., Estève D., Fourniols J.-Y. (2009). Smart homes-current features and future perspectives. *Maturitas*.

[17] Chandel V., Sinharay A., Ahmed N., Ghose A. Exploiting IMU sensors for IoT enabled health monitoring.

[18] Singh R. (2014). A proposal for mobile e-care health service system using IoT for Indian scenario. *Journal of Network Communications and Emerging Technologies (JNCET)*.

[19] Pinto S. J. We-care: an IoT-based health care system for elderly people.

[20] Huang C. H., Cheng K. W. (2014). RFID technology combined with IoT application in the medical nursing system. *Bulletin of Networking, Computing, Systems, and Software*.

[21] Cocco J. (2011). Smart home technology for the elderly and the need for regulation. *Pittsburgh Journal of Environmental & Public Health Law*.

[22] Papageorgiou E. I. Fuzzy cognitive map-based decision support system for thyroid diagnosis management.

[23] Darshan K. R. A comprehensive review on the usage of Internet of Things (IoT) in healthcare system.

[24] Tsafack N., Sankar S., Abd-El-Atty B. (2020). A new chaotic map with dynamic analysis and encryption application in internet of health things. *IEEE Access*.

[25] Abd-El-Atty B., Iliyasu A. M., Alaskar H., Abd El-Latif A. A., Ahmed A. (2020). A robust quasi-quantum walks-based steganography protocol for secure transmission of images on cloud-based E-healthcare platforms. *Sensors*.

[26] Abd EL-Latif A. A., Abd-El-Atty B., Venegas-Andraca E. M., Andraca S. E. V. (2020). Controlled alternate quantum walks based privacy preserving healthcare images in Internet of Things. *Optics & Laser Technology*.

[27] Vippalapalli V. A. Internet of things (IoT) based smart health care system.

[28] Fischer M. A. From books to bots: using medical literature to create a chatbot.

[29] Ghose A. E. UbiHeld: ubiquitous healthcare monitoring system for elderly and chronic patients.

[30] Gope P. A. (2015). BSN-Care: a secure IoT-based modern healthcare system using body sensor network. *IEEE Sensors Journal*.

[31] Kumar K. S. IoT based health monitoring system for autistic patients.

[32] Matar G. E. Internet of Things in sleep monitoring: an application for posture recognition using supervised learning.

[33] Moore L. (1999). Measuring quality and effectiveness of prehospital EMS. *Prehospital Emergency Care*.

[34] Otoom A. F. (2015). Effective diagnosis and monitoring of heart disease. *International Journal of Software Engineering and Its Applications*.

[35] Parthiban G., Srivatsa K. (2012). Applying machine learning methods in diagnosing heart disease for diabetic patients. *International Journal of Applied Information Systems*.

[36] Puri C. E. ICarMa: inexpensive cardiac Arrhythmia management-an IoT healthcare analytics solution.

[37] Sen S. K. (2014). Application of meta-learning algorithms for the prediction of diabetes disease. *International Journal of Advanced Research in Computer Science and Management Studies*.

[38] Qin F. Y., Lv Z. Q., Wang D. N., Hu B., Wu C. (2020). Health status prediction for the elderly based on machine learning. *Archives of Gerontology and Geriatrics*.

[39] Tang H., Yao E., Tan G., Guo X. A fast and accurate 3D fine-tuning convolutional neural network for alzheimer’s disease diagnosis.

[40] Abrol A., Bhattarai M., Fedorov A., Du Y., Plis S., Calhoun V. (2020). Deep residual learning for neuroimaging: an application to predict progression to Alzheimer’s disease. *Journal of Neuroscience Methods*.

[41] Dimitriadis S. I., Liparas D., Tsolaki M. N. (2018). Random forest feature selection, fusion and ensemble strategy: combining multiple morphological MRI measures to discriminate among healthy elderly, MCI, cMCI and alzheimer’s disease patients: from the alzheimer’s disease neuroimaging initiative (ADNI) database. *Journal of Neuroscience Methods*.

[42] Liu S., Liu S., Cai W., Pujol K., Kikinis R., Feng D. Early diagnosis of Alzheimer’s disease with deep learning.

[43] Lu D., Popuri K., Ding G. W., Balachandar R., Beg M. F. (2018). Multimodal and multiscale deep neural networks for the early diagnosis of Alzheimer’s disease using structural MR and FDG-PET images. *Scientific Reports*.

